# The Limited English Proficient Population: Describing Medicare, Medicaid, and Dual Beneficiaries

**DOI:** 10.1089/heq.2017.0036

**Published:** 2018-05-01

**Authors:** Kimberly Proctor, Shondelle M. Wilson-Frederick, Samuel C. Haffer

**Affiliations:** ^1^ Centers for Medicare and Medicaid Services, Centers for Medicaid and CHIP Services, Baltimore, Maryland.; ^2^ Centers for Medicare and Medicaid Services, Office of Minority Health, Baltimore, Maryland.; ^3^ U.S. Equal Employment Opportunity Commission, Washington, District of Columbia.

**Keywords:** estimation, health disparities, limited English proficient (LEP), Medicaid, Medicare

## Abstract

**Purpose:** The Limited English Proficient (LEP) population experiences well-documented suboptimal health outcomes and substandard provider experiences. The lack of national estimates on the size of the LEP population relative to the healthcare setting makes examining health outcomes for this population very difficult. This analysis addresses this limitation by publishing population estimates for LEP persons enrolled in Medicare, Medicaid, and Duals (enrolled in Medicare and Medicaid). Focusing on the Medicare and Medicaid programs provides an important foundation as these programs are not only the largest insurers in the United States but are also governed by legislation that requires LEP persons to receive equitable access to care.

**Methods:** Data from the 2014 American Community Survey Public Use Microdata Sample (ACS PUMS) were used to produce national estimates and measures of statistical accuracy for the LEP population enrolled in Medicare and/or Medicaid (LEPMM).

**Results:** In 2014, there were approximately 8.7 million LEP persons enrolled in Medicare, Medicaid, or both programs (Duals). The LEPMM was concentrated along the western and eastern coastlines and the southwestern region, with California and New York each containing more than 1 million LEPMMs. The LEPMM was also highly diverse with varying disability status, and most were racial or ethnic minorities and elderly.

**Conclusion:** These findings provide a foundation for measuring an understudied and at-risk population that will enable population health professionals to develop effective culturally and linguistically, and appropriate services and policies that address health disparities in the LEPMM.

## Introduction

In 2013, 25.5 million people (8.5% of the U.S. population) reported that they were Limited English Proficient (LEP), or spoke a language other than English at home and spoke English “not very well,” “not well,” or “not at all.” Since 1990, the LEP population has increased by 80%.^1^ The size and growth of the LEP population are particularly relevant for healthcare providers, as 80% of providers encounter LEP patients throughout the year and 43% encounter them daily.^2^

LEP status is linked to multiple suboptimal health outcomes,^3,4^ such as higher rates of disability,^5^ poor self-rated health,^5–9^ higher rates of psychological distress and mental illness,^5^ and lower rates of visiting doctors or having a regular healthcare provider.^2,6,9–13^ Moreover, these health outcomes are strongly related to self-reported difficulty communicating and comprehending medical information from providers, including challenges with understanding written information at the doctor's office and reading prescription bottles.^5,14^ LEP patients also face a complex provider environment that often lacks interpreter services or appropriate health education materials.^15,16^ These structural and clinical challenges often result in diminished patient satisfaction, postcare adherence, patient safety, and lack of equity in the provision of healthcare.^5,14^ Given that 75% of LEP individuals are between the ages of 18–64 years (nonelderly), 15% are 65 and over (elderly),^1^ and more than 34% are insured by Medicare and/or Medicaid, there is a greater need to understand the health needs of this population and have accurate and reliable estimates of the number of LEP persons enrolled in Medicare and/or Medicaid (LEPMM).

In 2013, Medicare and Medicaid programs constituted the largest insurers in the United States, covering over 109 million combined beneficiaries.^17^ The health inequities facing LEP persons are especially problematic because the Centers for Medicare & Medicaid Services (CMS), which administers Medicare and Medicaid health insurance programs, is obligated to assure equitable care to all beneficiaries. Beginning with Title VI of the Civil Rights Act of 1964, federal law prohibits discrimination based on national origin, including language preference, with specific regulations banning discrimination in the provision of Medicare and Medicaid.^18^ Recent policies have further entrenched language access with a focus on the rights of LEP persons. The National CLAS Standards (2013) was developed with the goal of guaranteeing access to culturally and linguistically appropriate services for LEP beneficiaries^19^ and the Medicare Improvements for Patients and Providers Act of 2008 demonstrates a continued legal commitment to guaranteeing language access and evaluating provider compliance. Overall, these policies demonstrate that LEP beneficiaries are an important subpopulation for CMS and the agency is legally required to protect their rights in the healthcare setting.

Despite the increasing growth of the LEP population, CMS has limited information on the language preferences and needs of its beneficiary population. This analysis addresses this gap in the literature by using the American Community Survey (ACS) Public Use Microdata Sample (PUMS) to provide the first estimates of the size of the LEP population enrolled in Medicare and/or Medicaid population, with the goal of enhancing LEP-focused health policy and research.

## Methods

To enumerate and describe the LEPMM, this analysis uses the PUMS files from the U.S. Census Bureau, 2014 ACS 1-year files. The ACS is an ongoing survey of the U.S. population that collects data on topics such as language preference and health insurance status, and is the primary source for limited English proficiency estimates used by the U.S. Department of Health and Human Services.^20,21^

For this analysis, ACS PUMS data were used to identify persons who reported speaking a language other than English at home, spoke English less than very well, and reported enrollment in Medicare, Medicaid, or both (Duals). Hispanic ethnicity included persons of any racial group who reported Hispanic ethnicity. This analysis includes non-Hispanic members of the following racial groups: White, Black, Asian, American Indian or Alaska Native (AIAN), Other, and Multiracial. The ACS collects information on six types of difficulty related to disability: hearing, visual, cognitive, ambulatory, self-care, and independent living. Respondents reporting any one of these disability types were classified as having a disability. This analysis excludes unknown, illegible, N/A, or blank responses, which impact the estimates, as data on the LEP population contain a high degree of missingness.

Due to missing information on LEP status in the 2014 ACS PUMS, estimates regarding LEP status are missing for nearly 9 million members of the Medicare, Medicaid, and/or Duals population. Therefore, it is possible that this analysis significantly underestimates the size of the LEP population. This may impact the Medicaid program most substantially, as 98.6% of all persons' missing information regarding their LEP status reported enrollment in Medicaid. Medicare (0.7%) and Duals (0.6%) had substantially lower rates of missingness. Consequently, readers should interpret these estimates as conservative estimates that represent a lower bound of the LEPMM, particularly regarding the Medicaid program.

Because the ACS is a sample of the U.S. population, the estimates produced using the PUMS data also contain sampling error. To ensure that appropriate conclusions are drawn about the quality of the data, the standard error (SE), the margin of error (MOE), and the coefficient of variation (CV)^22^ are also reported with the population estimates. These statistics allow for the measurement of statistical reliability and provide valuable information regarding the usability of the estimates for the LEPMM. The sampling error is directly related to sample size and is expected to increase as the sample size decreases and vice versa; thus the sample size accompanies all estimates in this analysis.

In the context of the ACS PUMS data, the SE measures the variability of the estimate due to sampling^22^ and demonstrates the extent to which the population estimate deviates from the true population value.^23^ The MOE is used to calculate the confidence intervals (CIs), which represent the range that is expected to contain the average value of the estimate, given the level of confidence. For interpretation purposes, the 90% confidence level indicates that we should expect the true population size to fall between the upper and lower CIs in 9 of 10 samples. The lower CI is calculated by subtracting the MOE from the estimate, while the upper CI is calculated by adding the MOE to the estimate. Finally, the CV measures the relative amount of sampling error that accompanies an estimate and reflects the ratio of the SE to the estimate itself.^24^ Because it is expressed as a percent, it is useful in understanding the overall validity of an estimate compared to other estimates. As the CV approaches 0, the reliability of the estimate increases and provides evidence that the estimate is accurate; as the CV approaches 100, the reliability of the estimate decreases and indicates that the estimate may not be accurate or interpretable.

## Results

Table 1 contains estimates of the size of the LEPMM in 2014, along with the corresponding measures of statistical accuracy. There were approximately 8.7 million LEPMMs in 2014, with the majority (52.9%) enrolled in Medicaid, followed by Medicare (26.0%) and Duals (21.1%). Overall, the LEPMM comprised 8.4% of the total Medicare, Medicaid, and Dual population in 2014.

**Table 1.** **Estimates of the Limited English Proficient in Medicare and/or Medicaid Population, ACS PUMS 2014**

	Number of LEP Persons (*%*)	Sample Size	SE	MOE	CV
Medicaid	4,583,162 (52.9)	38,211	21,058	34,641	0.46
Medicare	2,252,783 (26.0)	23,062	17,856	29,373	0.79
Duals	1,835,707 (21.1)	17,426	17,010	27,982	0.93
Total	8,671,652	78,699	45,545	74,921	0.53

ACS, American Community Survey; CI, confidence interval; CV, coefficient of variation; PUMS, public use microdata sample; MOE, margin of error.

Table 2a highlights the racial composition of the LEPMM. The majority of LEPMMs are racial and ethnic minorities, with the greatest representation from Hispanics. The Medicaid program insures the most diverse population, with 9.5% of LEP persons enrolled in Medicaid self-identifying as white and a majority of the Medicaid LEP population self-identifying as Hispanic (65.6%). Among Duals, 15.9% of LEP persons were white and more than half were classified as Hispanic (53.4%). For Medicare LEP beneficiaries, 22.3% were white and less than half identified as Hispanic (47.6%). In total, the LEPMM includes more than 7.4 million racial and ethnic minorities (data not shown).

**Table 2.** **Demographic and Health Characteristics of the Limited English Proficient in Medicare and/or Medicaid Population, ACS PUMS 2014**

2a.Racial^*^ and Ethnic Composition of the LEPMM,% Total LEPMMs								
	White	Black	AIAN	Asian	Other	Multiracial	Hispanic	Total
Medicaid	9.5	4.6	0.4	18.5	0.5	0.9	65.6	100
Medicare	22.3	2.5	0.4	26.1	0.2	0.8	47.6	100
Duals	15.9	3.0	0.6	26.1	0.2	0.9	53.4	100
^*^ Non-Hispanic racial groups are shown.								
**2b. Age Distribution of the LEPMM,% Total LEPMMs**								
	0–18 years	19–64 years	≥65 years	Total				
Medicaid	31.1	68.9	0.0	100				
Medicare	0.6	11.2	88.2	100				
Duals	0.6	13.7	85.7	100				
**2c.Educational Attainment of the LEPMM,% Total LEPMMs**								
	<High School	High School Diploma	≥Bachelor's degree	Total				
Medicaid	63.7	30.7	5.7	100				
Medicare	48.2	36.7	15.1	100				
Duals	61.5	28.7	9.9	100				
**2d. Disability Status of the LEPMM,% Total LEPMMs**								
	Not Disabled	Disabled	Total					
Medicaid	87.2	12.8	100					
Medicare	65.4	34.6	100					
Duals	41.1	58.9	100					

Table 2b displays the age distribution of the LEPMM. Medicare insures persons 65 years and older, those covered by Social Security Disability Insurance (SSDI), or with end stage-renal disease; Medicaid insures low-income persons, children and families, pregnant women, and people with disabilities; and Duals are enrolled in both programs. Table 2b shows that the majority of LEP persons enrolled in Medicaid were nonelderly (≤18 years, 31.1% and 19–64 years, 68.9%), whereas the majority of LEP Duals (85.7%) and those enrolled in Medicare (88.2%) were elderly. Therefore, LEP persons enrolled in Medicaid were substantially younger than their Medicare and dually enrolled counterparts.

Table 2c shows varying levels of educational attainment among LEP persons by federally insured program. Among LEP persons enrolled in Medicaid, a majority (63.7%) completed less than high school and 5.7% had earned a Bachelor's degree or greater. However, less than half (48.2%) of LEP Medicare beneficiaries completed less than high school and 15.1% had earned a Bachelor's degree or greater. Among LEP Duals, a majority (61.5%) completed less than high school and 9.9% earned a Bachelor's degree or greater.

As shown in Table 2d, the prevalence of disability varied by federal insurance status among LEPMMs. LEP Duals had the highest prevalence (58.9%) of disability, followed by LEP Medicare beneficiaries (34.6%) and LEP persons enrolled in Medicaid (12.8%).

Table 3 displays population estimates for 10 states with the largest LEPMM, along with the sample size, SE, MOE, and CV. These findings indicate that the largest LEPMM resides in California, comprising over 2.6 million LEPMM persons (30.5% of the total LEPMM), followed by New York, which includes over 1.2 million LEPMM persons (14.3% of the total LEPMM). The LEPMM is heavily concentrated in a few states, as 81.6% of the total LEPMM resides in the ten states. Given the high degree of accuracy surrounding the estimates, with the least precise estimate having a CV of 4.55 (Pennsylvania) and the most precise having a CV of less than 1 (California), these data are highly reliable.

**Table 3.** **Ten Largest Medicare- and/or Medicaid-Insured Limited English Proficient Populations by State, ACS PUMS 2014**

State	Number of LEPMMs (%)	Sample Size	SE	MOE	CV
California	2,646,080 (30.5)	25,797	17,996	29,603	0.68
New York	1,242,798 (14.3)	10,666	14,541	23,920	1.17
Texas	950,041 (11.0)	8,807	13,513	22,229	1.42
Florida	737,963 (8.5)	6,731	9,837	16,182	1.33
Massachusetts	331,719 (3.8)	2,864	6,978	11,479	2.1
Illinois	314,087 (3.6)	2,609	9,002	14,809	2.87
New Jersey	302,527 (3.5)	2,779	7,428	12,219	2.46
Arizona	215,958 (2.5)	2,278	6,106	10,044	2.83
Pennsylvania	170,676 (2.0)	1,151	7,765	12,773	4.55
Washington	162,956 (1.9)	1,396	6,312	10,384	3.87
Total	7,074,805 (81.6)	65,078	36,263	59,653	0.51

**FIG. 1.**
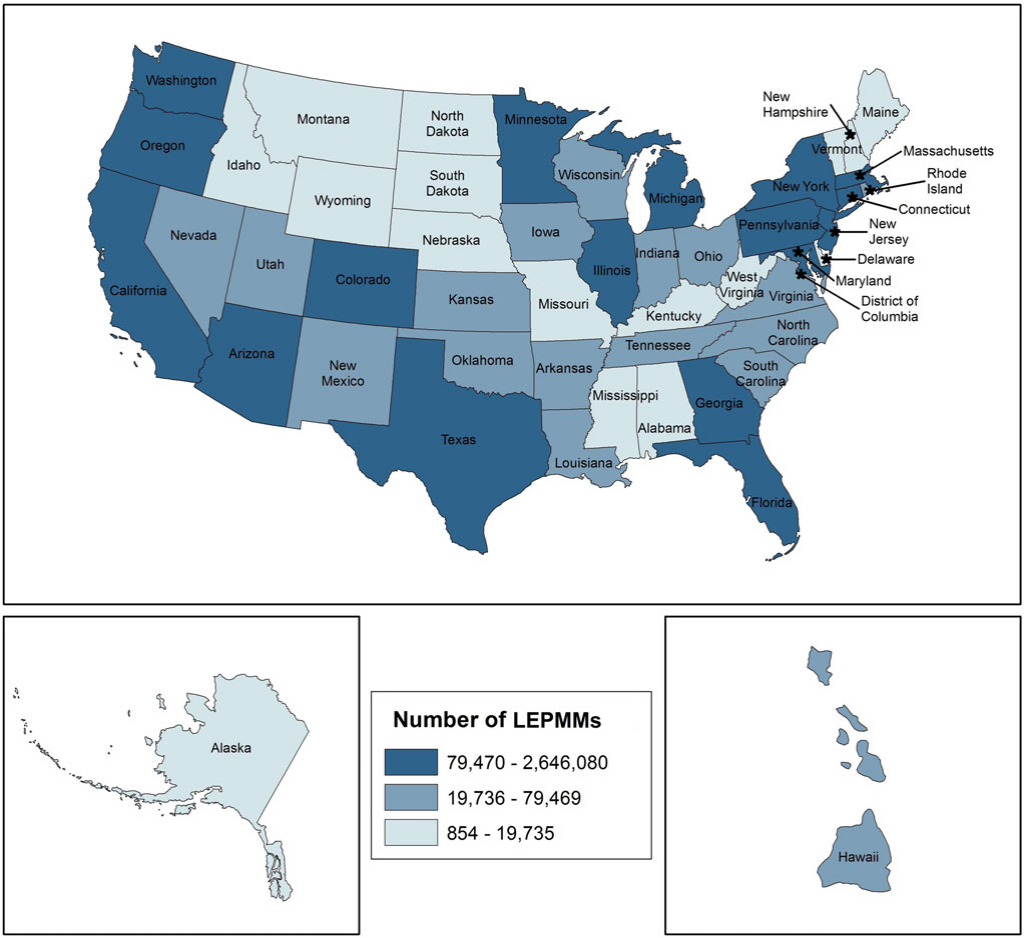
Geographic distribution of the LEPMM in the United States. LEPMM, Limited English Proficient in Medicare and/or Medicaid.

Figure 1 illustrates that the LEPMM is primarily located along the East and West Coasts and in the Southwest. This map also shows that Midwestern states of Minnesota, Illinois, and Michigan include LEPMM ranging 79,470–2,646,080 people. The map includes a hatched overlay representing potentially unreliable estimates, (states with CVs greater than 15) and 11 states meet these criteria: Alaska, Idaho, Maine, Mississippi, Montana, New Hampshire, North Dakota, South Dakota, Vermont, West Virginia, and Wyoming. For these relatively imprecise estimates, the CV ranged from a low of 16.3 in Maine to a high of 42.7 in Montana. The average CV across all unreliable estimates was 23.3, indicating that readers should interpret these estimates with caution.

The ACS determines a respondent's preferred language by asking if they speak a language other than English at home and, if so, having them write-in their preferred language. Based on the 2014 ACS, there were over 100 language groups. Table 4 contains population estimates for language groups that contained 50,000 or more LEPMMs. These languages contain over 90.0% of the total LEPMM. In 2014, the 10 largest language groups were: Spanish, Chinese, Vietnamese, Russian, Arabic, Filipino/Tagalog, Korean, Cantonese, French or Haitian Creole, and Italian. These language groups contain over 80% of the total LEPMM. In addition, the error surrounding these estimates falls within conventionally accepted ranges, indicating that they are reliable and accurate depictions of language diversity among LEPMM persons. Of the 109 languages spoken among LEPMMs, 62 have CVs that fall above conventional levels of reliability and are potentially unreliable.

**Table 4.** **Languages with 50,000 or More Limited English Proficient in Medicare and/or Medicaid Speakers, ACS PUMS 2014**

Language	Number of LEPMMs (%)	Sample Size	SE	MOE	CV
Spanish	5,182,495 (59.8)	45,242	19,338	31,811	0.4
Chinese	379,772 (4.4)	3,829	8,598	14,144	2.3
Vietnamese	325,611 (3.8)	3,162	9,160	15,068	2.8
Russian	192,971 (2.2)	1,819	5,984	9,843	3.1
Arabic	189,505 (2.2)	1,531	8,131	13,376	4.3
Filipino, Tagalog	186,331 (2.2)	1,935	5,295	8,710	2.8
Korean	177,443 (2.1)	1,753	6,053	9,957	3.4
Cantonese	141,577 (1.6)	1,318	6,018	9,900	4.3
French or Haitian Creole	119,475 (1.4)	970	5,322	8,754	4.5
Italian	108,176 (1.3)	1,196	3,767	6,197	3.5
Portuguese	92,250 (1.1)	894	5,111	8,408	5.5
French	80,223 (0.9)	921	3,757	6,180	4.7
Persian, Iranian, Farsi	78,058 (0.9)	646	4,170	6,860	5.3
Mandarin	72,332 (0.8)	650	4,943	8,131	6.8
Polish	68,441 (0.8)	686	3,697	6,081	5.4
Bengali	64,647 (0.8)	588	4,421	7,272	6.8
Armenian	61,854 (0.7)	533	4,279	7,039	6.9
German	59,918 (0.7)	743	2,933	4,824	4.9
Japanese	57,983 (0.7)	652	3,451	5,677	6.0
Urdu	57,058 (0.7)	498	4,044	6,653	7.1
Miao, Hmong	54,950 (0.6)	400	5,215	8,579	9.5
Yiddish, Jewish	52,873 (0.6)	626	3,843	6,322	7.3
Panjabi	51,628 (0.6)	416	3,748	6,165	7.3

## Discussion

Knowing the size, geographic location, and language group characteristics of the federally insured population is important for complying with federal policy and evaluating federal programs. However, due to limitations in data collection, CMS currently lacks accurate population estimates that reflect the size and diversity of its beneficiary population. This analysis addresses this gap by using ACS PUMS data to estimate the size of the LEPMM and describe its demographic and health characteristics.

In 2014, with a population totaling almost 8.7 million,^25^ LEPMMs comprised a meaningful portion of the overall Medicare, Medicaid, and dually enrolled population. In addition to the myriad laws, regulations, and policies demonstrating the importance of the LEPMM, the sheer size (representing 8.4% of all CMS beneficiaries) indicates that providers are increasingly likely to encounter LEPMM persons. This research revealed that LEPMM persons tend to reside along the western and eastern coastlines, southwestern region, and in midwestern states of Illinois, Michigan, and Minnesota. Examining the demographic and health features of the LEPMM showed that a majority of LEPMMs were racial or ethnic minorities, aged 65 and older, and had lower levels of educational attainment with Dual LEPs reporting the highest prevalence of disability. Among federally insured LEP persons, our study showed that 50,000 or more spoke 19 different languages.

To ensure equity in healthcare, it will be necessary for providers and healthcare systems to provide culturally and linguistically appropriate services for their LEP patients. A recent study by Lee et al. (2017) showed that among Chinese- and Spanish-speaking patients, a bedside interpreter phone system intervention was associated with greater informed consent; however, a disparity was still observed regarding the care of LEP patients.^26^ Evidence has shown a reduction in readmission rates and expenditures when hospitals provide convenient access to interpreters.^27^ In addition, dosing errors among Hispanic parents, who may be LEP or have limited health literacy, have been shown to be problematic; however, the ability to better characterize the LEP population may assist in reducing these errors.^28^ Also, providing language-concordant care (i.e., Spanish-speaking providers caring for Spanish-speaking patients) may be a feasible solution to thwart health disparities experienced by LEP patients.^29^

Overall, results from this study will enable providers to better serve their LEP patients. Providers located along the East Coast, West Coast, and Southwest should expect to have the majority of LEP patient encounters, given that over 80% of the entire LEPMM resides in these regions. In addition, providers should be aware that nearly 60% of all LEPMMs speak Spanish as their primary language; therefore, it might be beneficial to focus on Spanish language translation services in the geographic regions densely occupied by LEPMMs.

Using ACS PUMS data was a major strength of the study. These data allowed for the generation of reliable and national estimates of the LEPMM. To our knowledge, this is the first time that national estimates, along with measures of statistical accuracy, have been produced on the U.S. LEPMM. This foundation will help support the provision of culturally and linguistically appropriate services for the LEPMM. However, the ACS PUMS data do not capture information on respondents preferred language to obtain health information. Therefore, we were unable to gauge the health literacy level of the study population and this should be examined in future research. As the U.S. population continues to become more diverse, it is critical to have national data sources that provide this information. Even with these limitations, the estimates described herein will inform policy decisions regarding the health needs of one of the most at-risk and growing federally-insured populations.

## Conclusion

LEP persons represent a sizable and meaningful portion of the overall Medicare**,** Medicaid, and dually enrolled populations. In the CMS Strategic Language Access Plan,^21^ the goal is to ensure that 90% of beneficiaries requesting LEP-related materials and/or assistance receive it during the first attempt and they are satisfied with the customer service provided at least 80% of the time. However, without knowing which languages are most likely to be encountered, it is very difficult for providers to accurately prepare for language requests. Therefore, accurate population estimates of language group by program are essential to the implementation of federal laws and policies aimed at enumerating and servicing the LEPMM. These study findings move federal planning and provider efficiency toward the ultimate goal of providing equitable access to healthcare across multiple language groups. With demographic data describing where LEPMM persons live and which languages they speak, both the government and healthcare providers can operate more effectively by strategically targeting those areas with the greatest degree of language need.

## Abbreviations Used

ACS: American Community Survey
AIAN: Asian, American Indian, or Alaska Native
CI: confidence interval
CMS: Centers for Medicare & Medicaid Services
CV: coefficient of variation
LEP: limited English proficient
LEPMM: limited English proficient in medicare and/or medicaid
MOE: margin of error
PUMS: public use microdata sample
SE: standard error

## References

[1] Zong J, Batalova J The Limited English Proficient Population in the United States. Washington, DC: Migration Policy Institute, 2015

[2] Kang SY, Howard D, Kim J, et al. English language proficiency and lifetime mental health service utilization in a national representative sample of Asian Americans in the USA. J Public Health (Oxf). 2010;32:431–439 20202979 10.1093/pubmed/fdq010 PMC2924787

[3] Paredes AZ, Idrees JJ, Beal EW, et al. Influence of english proficiency on patient-provider communication and shared decision-making. Surgery. 2018 [Epub ahead of print]; DOI: 10.1016/j.surg.2018.01.012 29482884

[4] Kim EJ, Kim T, Paasche-Orlow MK, et al. Disparities in Hypertension Associated with Limited English Proficiency. J Gen Int Med. 2017;32:632–639 10.1007/s11606-017-3999-9 PMC5442015 28160188

[5] Kim G, Worley CB, Allen RS, et al. Vulnerability of older Latino and Asian immigrants with limited English proficiency. J Am Geriatr Soc. 2011;59:1246–1252 21718269 10.1111/j.1532-5415.2011.03483.x

[6] DuBard CA, Gizlice Z Language spoken and differences in health status, access to care, and receipt of preventive services among US Hispanics. Am J Public Health. 2008;98:2021–2028 18799780 10.2105/AJPH.2007.119008 PMC2636430

[7] Flores G The impact of medical interpreter services on the quality of health care: a systematic review. Med Care Res Rev. 2005;62:255–299 15894705 10.1177/1077558705275416

[8] Ponce NA, Hays RD, Cunningham WE Linguistic disparities in health care access and health status among older adults. J Gen Int Med. 2006;21:786–791 10.1111/j.1525-1497.2006.00491.x PMC1924691 16808783

[9] Shi L, Lebrun LA, Tsai J The influence of English proficiency on access to care. Ethn Health. 2009;14:625–642 19953393 10.1080/13557850903248639

[10] Derose KP, Baker DW Limited English proficiency and Latinos' use of physician services. Med Care Res Rev. 2000;57:76–91 10705703 10.1177/107755870005700105

[11] Brotanek JM, Weitzman M, Halterman J, et al. Inadequate access to care among children with asthma from Spanish-speaking families. J Health Care Poor Underserved. 2005;16:63–73 15741710 10.1353/hpu.2005.0005

[12] Jacobs EA, Karavolos K, Rathouz PJ, et al. Limited English proficiency and breast and cervical cancer screening in a multiethnic population. Am J Public Health. 2005;95:1410–1416 16043670 10.2105/AJPH.2004.041418 PMC1449374

[13] Pippins JR, Alegría M, Haas JS Association between language proficiency and the quality of primary care among a national sample of insured Latinos. Med Care. 2007;45:1020 18049341 10.1097/MLR.0b013e31814847be PMC2836911

[14] Wilson E, Chen AH, Grumbach K, et al. Effects of limited English proficiency and physician language on health care comprehension. J Gen Intern Med. 2005;20:800–806 16117746 10.1111/j.1525-1497.2005.0174.x PMC1490205

[15] Morales LS, Cunningham WE, Brown JA, et al. Are Latinos less satisfied with communication by health care providers? J Gen Intern Med. 1999;14:409–417 10417598 10.1046/j.1525-1497.1999.06198.x PMC1496614

[16] Betancourt JR, Green AR, Carrillo JE, et al. Defining cultural competence: a practical framework for addressing racial/ethnic disparities in health and health care. Public Health Rep. 2003;118:293–302 12815076 10.1016/S0033-3549(04)50253-4 PMC1497553

[17] Sisko AM, Keehan SP, Cuckler GA, Madison AJ, Smith SD, Wolfe CJ, et al. National health expenditure projections, 2013–23: faster growth expected with expanded coverage and improving economy. Health Affairs. 2014;33:1841–1850. Accessed on 218, 2015 25187525 10.1377/hlthaff.2014.0560

[18] Executive Order 13166: Improving Access to Services for Persons with Limited English Proficiency 2000 Available at www.lep.gov/13166/eo13166.html Accessed on 218, 2015

[19] U.S. Department of Health and Human Services-Office of Minority Health. Enhanced National CLAS Standards 2013 Available at https://www.thinkculturalhealth.hhs.gov/clas/standards Accessed on 130, 2018

[20] U.S. Department of Health and Human Services-Office of the Inspector General. Guidance and Standards on Language Access Services: Medicare Providers. Washington, DC: U.S. Department of Health and Human Services, 2010

[21] U.S. Department of Health and Human Services-Centers for Medicare & Medicaid Services. Strategic Language Access Plan (LAP): To Improve Access to CMS Federally Conducted Activities by Persons with Limited English Proficiency (LEP), 2014 Updates. Baltimore, MD: Centers for Medicare & Medicaid Services, 2014

[22] U.S. Census Bureau. A Compass for Understanding and Using American Community Survey Data: What State and Local Governments Need to Know. Washington, DC: U.S. Government Printing Office, 2009

[23] U.S. Census Bureau. Things that May Affect the Estimates from the American Community Survey. Washington, DC: U.S. Government Printing Office, 2013

[24] Evans MJ, Rosenthal JS Probability and Statistics: The Science of Uncertainty. New York, NY: W.H. Freeman and Company, 2004

[25] Centers for Medicare & Medicaid-Office of Minority Health. Understanding Communication and Language Needs of Medicare Beneficiaries 2017 Available at www.cms.gov/About-CMS/Agency-Information/OMH/Downloads/Issue-Briefs-Understanding-Communication-and-Language-Needs-of-Medicare-Beneficiaries.pdf Accessed on 612, 2017

[26] Lee JS, Pérez-Stable EJ, Gregorich SE, et al. Increased access to professional interpreters in the hospital improves informed consent for patients with limited English proficiency. J Gen Int Med. 2017;32:863–870 10.1007/s11606-017-3983-4 PMC5515780 28185201

[27] Karliner LS, Pérez-Stable EJ, Gregorich SE Convenient access to professional interpreters in the hospital decreases readmission rates and estimated hospital expenditures for patients with limited english proficiency. Med Care. 2017;55:199–206 27579909 10.1097/MLR.0000000000000643 PMC5309198

[28] Harris LM, Dreyer BP, Mendelsohn AL, et al. Liquid medication dosing errors by hispanic parents: role of health literacy and english proficiency. Acad Pediatr. 2017;17:403–410 28477800 10.1016/j.acap.2016.10.001 PMC5424611

[29] Parker MM, Fernández A, Moffet HH, et al. Association of patient-physician language concordance and glycemic control for limited–english proficiency latinos with type 2 diabetes. JAMA Int Med. 2017;177:380–387 10.1001/jamainternmed.2016.8648 PMC5339062 28114680

